# Illuminating “the dark side of the HBB”: Visualizing the full spectrum of Cas9 outcomes

**DOI:** 10.1016/j.omtn.2026.102920

**Published:** 2026-04-15

**Authors:** G. Scalisi, M. Laurent, M. Amendola

**Affiliations:** 1 Genethon, 91000 Evry, France; 2 Integrare Research Unit UMR_S951, Université Paris-Saclay, University Evry, Inserm, Genethon, Evry, France; 3 Department of Clinical and Experimental Medicine, University of Foggia, Foggia, Italy

## Main text

Sickle cell disease (SCD) is one of the most prevalent and severe inherited monogenic disorders caused by a point mutation that results in a glutamic acid-to-valine substitution at position 6 of the β-globin (HBB) chain. This sickle β-globin variant (β^S^) combines with α-globin chains to form sickle hemoglobin (HbS), which polymerizes under low oxygen conditions, causing red blood cells (RBCs) sickling, reduced deformability, hemolysis, recurrent vaso-occlusive crises, and progressive organ damage, ultimately causing poor quality of life and shortened life expectancy.^1^

To overcome this unmet medical need, innovative autologous hematopoietic stem cell (HSC) gene therapy approaches have been developed using CRISPR-Cas9. In 2023, this technology led to the approval of Casgevy, the first clinically approved CRISPR-Cas9-based treatment, which induces fetal hemoglobin (HbF) through the disruption of the BCL11A erythroid enhancer.^2^

Although increasing HbF has a therapeutic benefit, it does not reduce the amount of β^S^-globin, which competes for α-globin chain availability and hemoglobin formation. For this reason, strategies aimed at directly correcting the *HBB* point mutation remain highly attractive because they could restore physiological globin chain stoichiometry. Several gene correction strategies relying on Cas9 ribonucleoproteins (RNPs) together with ssODN^3^ or AAV6^4^ donors have entered preclinical and early clinical development.^5^

However, because homology directed repair (HDR) efficiency remains intrinsically low in HSCs, a substantial proportion of edited alleles undergo unintended genomic modifications, including in-frame edits, frameshifts, large deletions (>200 bp), insertions, chromosomal truncations, and more complex rearrangements.^2^^,^^3^^,^^6^ Because many of these genotypes can impair erythroid maturation or long-term HSC fitness, there is a growing need to develop analytical systems capable of resolving and functionally characterizing the full spectrum of Cas9-induced outcomes. Standard short-read genotyping emphasizes HDR and small indels but systematically underestimates large deletions and structural rearrangements at *HBB*, precisely the classes of events now highlighted by regulatory guidance as critical for long-term safety assessment.^7^

Given that analyzing all these events in primary HSCs is technically challenging and time-consuming, in this issue of *Molecular Therapy Nucleic Acids*, Karsenty and colleagues address this need by engineering a dual-fluorescent HUDEP-2 reporter cell line to enable allele-specific tracking of HBB expression following CRISPR editing.

**Figure 1:**
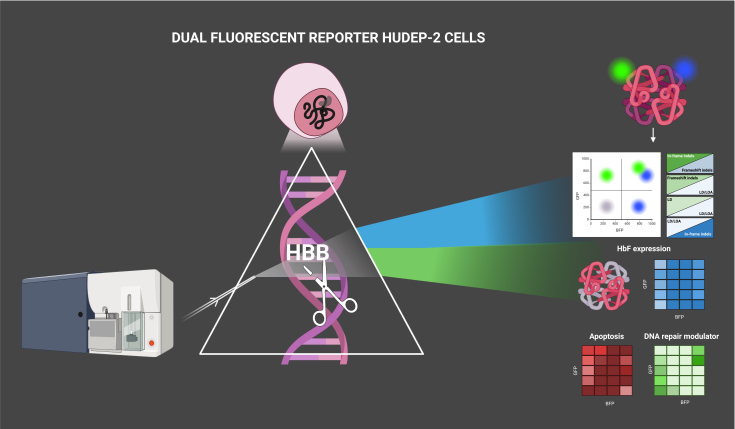
HUDEP-2 GFP/BFP reporter system This dual-color HUDEP-2 reporter system enables real-time visualization (by flow cytometer) of on-target repair outcomes following CRISPR-Cas9 editing (scissor) at the *HBB* locus. Indel-driven disruptions result in differential expression of GFP or BFP, producing distinct live-cell fluorescence states that directly mirror the underlying editing events. These readouts function as quantitative proxies to link genome-editing outcomes with downstream erythroid processes, including *HBB* transcriptional output, γ-globin (HbF) induction, maturation dynamics, and apoptosis and to assess the impact of DNA-repair modulators on editing profiles. This figure was created using Biorender.com.

Starting from a sickle HUDEP-2 cell line, the authors generated a biallelic P2A-GFP-pA cassette at the *HBB* 3′ end via HDR. One of the two GFP alleles was subsequently converted to BFP through a Y66H substitution, enabling each fluorescent protein to reflect gene transcription from its corresponding *HBB* allele. They then evaluated whether the GFP/BFP reporter system could correlate on-target DNA repair outcomes with live-cell fluorescence, transcriptional output, HbF induction, erythroid maturation, and apoptosis (Figure 1).^8^ Upon CRISPR-Cas9 editing with two clinically relevant gRNAs targeting *HBB* exon 1, cells reproducibly segregated into ∼20 distinct GFP/BFP fluorescence clusters, each capturing a characteristic genotypic class. By combining cell sorting with short-read next generation sequencing (NGS), long-read sequencing, ddPCR, and erythroid phenotyping, the authors constructed a detailed map linking each repair outcome, including in-frame edits, frameshift mutations with early or late nonsense codons, large deletions, and LOA (loss of allele) events, to transcription, protein output, HbF induction, differentiation potential, and apoptosis.

Several observations stand out. First, the system faithfully recapitulated the known consequences of frameshift indels in *HBB* exon 1: alleles introducing nonsense codons before position 19 partially escaped nonsense-mediated decay and thus exhibited intermediate expression, while those generating stop codons at or beyond position 19 underwent robust decay and displayed low fluorescence. Second, large deletions correlated with dim fluorescence, whereas LOA led to complete signal loss. Strikingly, LOA alleles, unlike frameshifted or promoter-deleted alleles, failed to induce HbF and instead triggered profound erythroid apoptosis, consistent with severe globin imbalance or disruption of regulatory elements within the β-globin locus, potentially affecting HBG1/2 regulation. These findings underscored LOA as a qualitatively distinct and potentially high-risk repair outcome. Interestingly, these unwanted alterations were strongly reduced in the presence of ssODN.

Finally, the platform also proved highly informative for evaluating the impact of pharmacological modulation of DNA repair pathways, commonly used to increase HDR efficiency, on editing and reporter expression.

Inhibition of non-homologous end joining (NHEJ) (via DNA-PK) or depletion of 53BP1 not only increased HDR but also increased LOA and large deletions, highlighting a potential trade-off between editing precision and genotoxicity. Conversely, microhomology-mediated end joining (MMEJ) (Polθ) inhibition reduced large deletions but paradoxically increased LOA, suggesting that LOA arose from repair pathways distinct from MMEJ. These results provide a cautionary note for HDR-boosting strategies: improvements measured by short-read NGS may be accompanied by clinically relevant increases in large deletions or chromosomal aberrations.

In addition, Karsenty’s platform enabled longitudinal assessment of cell fitness, maturation, chromatin structure, and transcriptional regulation as well as prospective isolation of live, genotype-enriched subpopulations for mechanistic work. HUDEP-2 reporter cells could be integrated with novel single-cell DNA sequencing approaches to further refine genotype-phenotype correlation and disentangle ambiguous categories, such as composite LOA events.^9^ This strategy could be extended to additional genomic loci relevant to SCD biology, other therapeutic targets, or other genome editing platforms, including base editors, which have been shown to induce large deletions,^10^ where linking repair profiles to functional consequences remains essential.

However, although HUDEP-2 cells offer the advantages of a tractable cell line model, they do not enucleate upon erythroid differentiation and differ from quiescent/slow-cycling HSCs in DNA repair pathway usage,^11^ proliferation state, chromatin context, and stress responses. In addition, the dual P2A-GFP/BFP tagging at both *HBB* alleles—implemented in a single clonal background—can alter β-globin protein structure and the local genomic environment, potentially perturbing 3D chromatin/LCR interactions and, thus, DSB repair. Finally, the authors tested only the SpCas9/ssODN combination; therefore, generalization to alternative editors or donor DNA for HDR remains uncertain.

Context from the broader literature suggests that *in vitro*-detectable on-target lesions at the HBG1/2 locus can become undetectable after long-term xenograft, consistent with negative selection against severely damaged clones in NGS mice.^9^ These observations do not diminish the central point of Karsenty’s study; rather, they clarify why LOA-type lesions, which Karsenty shows to be pro-apoptotic and HbF-silent, would be competitively purged *in vivo*, while still underscoring the value of pre-infusion detection and mitigation.

Ultimately, this work highlights the structural and functional diversity of on-target CRISPR editing at *HBB* and provides a robust framework for identifying genotypes that are therapeutically beneficial, neutral, or detrimental. By showing how subtle differences in repair outcomes propagate across transcription, globin stoichiometry, erythroid maturation, and cell survival, Karsenty and colleagues deliver a resource that will be indispensable for refining the efficacy and safety of *HBB*-targeted genome-editing strategies.

## Acknowledgments

M.A. receives research funding from the 10.13039/100011102European Union for the EDITSCD grant (101057659, https://editscd.eu/).

## Declaration of interests

All authors declare no competing interests.
